# Perceptual Balance, Stability, and Aesthetic Appreciation: Their Relations Depend on the Picture Type

**DOI:** 10.1177/2041669519856040

**Published:** 2019-06-12

**Authors:** Ronald Hübner, Martin G. Fillinger

**Affiliations:** Universität Konstanz, Germany

**Keywords:** aesthetics, preference, perceptual balance, complexity, perceptual stability

## Abstract

It is widely assumed that the aesthetic appreciation of a picture depends, among others, on how well the picture’s composition is perceptually balanced, where “perceptual balance” is often defined analogous to mechanics. To what extent this metaphor holds for different picture types, however, is still open. Therefore, in this study, we examined the relationship between balance, liking, and some objective measures with pictures from an aesthetic sensitivity test. These stimuli could be divided into single-element, multiple-element, and dynamic-pattern pictures. The results show that “balance” is interpreted differently, depending on the stimulus type. Whereas “mechanical” balance was applied to assess single-element pictures, the balance of multiple-element and dynamic-pattern pictures was rated more in the sense of gravitational stability. Only for the multiple-element stimuli, there was a positive relation between balance/stability and liking. Together, our findings show that there are different types of balance, and that their relation with liking depends on the picture type.

## Introduction

In art theory and related fields, it is widely assumed that the aesthetic appreciation of a picture depends, among others, on how well it is balanced (Arnheim, 1983; Bouleau, 2014; Kandinsky, 1926/1979; Ross, 1907). To define perceptual balance, *mechanical balance* is often used as metaphor. It is assumed that each element in a picture has a certain visual “weight” depending on its features like size, shape, and color (Arnheim, 1954). The weight exerts a corresponding perceptual force that increases with its distance from the picture’s “center of gravity.” Accordingly, a heavy weight located on one side of the fulcrum can be balanced by a lighter weight positioned further away on the other side. Usually, a picture is considered as balanced if all its elements are arranged in such a way that their perceptual forces are in equilibrium about a fulcrum and if the fulcrum coincides with the center of the picture (Locher, 2006).

By adopting the mechanical metaphor, the relative perceptual weight of pictorial elements can easily be assessed, at least within a simple context. One merely has to ask persons to adjust the horizontal position of a target element on a seesaw so that it is in equilibrium to a fixed element opposite to a fulcrum. This was one of the first methods applied in experimental research on perceptual balance (Pierce, 1894).

In recent years, even formal measures of perceptual balance have been developed based on the mechanical metaphor. One such measure is the *Assessment of Preference for Balance* (APB), developed by Wilson and Chatterjee (2005). These researchers assumed that the perceptual weight of each pixel in a picture is inversely related to its gray level, that is, dark pixels are heavier than bright ones. In addition, they divide a picture into four symmetrical areas around the horizontal, the vertical, and the two diagonal axes, respectively. The differences between the summed weights in opposite areas are then computed, and the mean of the eight differences is taken as the picture’s balance score. Wilson and Chatterjee (2005) have shown that APB scores not only can predict balance ratings but are also related to aesthetic appreciation.

A measure even more closely related to the mechanical metaphor is the *Deviation of the Center of “Mass”* (DCM) from the picture’s geometrical center (Hübner & Fillinger, 2016; McManus, Stöver, & Kim, 2011). Based on the pixels’ gray level, the center of perceptual mass (or the center of gravity) is computed analogously to mechanics. It is further assumed that the less this center deviates from the geometric center of the picture, the more the picture is perceived as balanced and liked. Hübner and Fillinger (2016) have shown that for the pictures used by Wilson and Chatterjee (2005), the DCM predicts balance and liking ratings similarly well as the APB. Averaged across the pictures, the DCM scores explained up to 68% of the variance for the balance ratings, and up to 86% of the variance for the liking ratings.

It should be noted, though, that these strong predictions are mainly due to the specific pictures applied in Hübner and Fillinger (2016) and in Wilson and Chatterjee (2005). They included only homogeneous elements with a simple shape and with an identical gray level. Such pictures have the advantage that balance can be varied strongly without affecting other characteristics. However, the obtained results say little about how far they can be generalized for more complex images. Some results (McManus et al., 2011; Thömmes & Hübner, 2018) suggest that the correlation between perceptual balance and aesthetic appreciation is much less for photographs. Thömmes and Hübner (2018), for instance, analyzed about 700 architectural photographs posted on Instagram by different photographers. For photographs representing a three-dimensional scene, they found that the scores (DCM and APB) correlated significantly with the number of Instagram Likes. However, the explained variance was only about 10%. Although this percentage is small, it must be taken into account that balance is only one of many factors usually determining aesthetic appreciation. Therefore, for more complex artwork, one cannot expect the same large correlations between balance and liking as for specifically constructed simple pictures.

Gershoni and Hochstein (2011) found that for Japanese calligraphies the APB even completely failed to predict perceptual balance ratings. Recently, Fillinger and Hübner (2018) replicated this result and observed similar negative results also for the DCM. However, they further showed that for these pictures perceptual balance ratings were completely unrelated with liking ratings.

Two conclusions can be drawn from these results. First, the absent relation between balance ratings and the balance measures indicates that persons sometimes apply concepts of perceptual balance that are not reflected by the APB and DCM measures. Fillinger and Hübner (2018), for instance, provided some evidence that under certain conditions balance is interpreted more in the sense of stability. Already Pierce (1894) observed that balance is mainly applied for the horizontal arrangement of elements, whereas for vertical arrangements stability plays a greater role. For instance, pictures were preferred when they had more weight in their lower part rather than in their upper half. Second, because the aesthetic appreciation of more complex pictures is usually determined by multiple factors, the effect of perceptual balance can be relatively small or even absent. In case these factors are known, it can be helpful to discount their effects. Fillinger and Hübner (2018), for instance, observed that prototypicality strongly determined the aesthetic appreciation of Japanese calligraphies. After taking this factor into account, the DCM showed again a significant relation with liking, but only for less prototypical calligraphies.

The aim of the present study was to further investigate the relations between the formal measures, perceptual balance, and aesthetic appreciation. For this objective, we used pictures from the *Visual Aesthetic Sensitivity Test* (VAST; Götz, 1981), developed by Götz, Borisy, Lynn, and Eysenck (1979). They consist of various configurations of different element types and, therefore, are more complex than those applied by Hübner and Fillinger (2016) and Wilson and Chatterjee (2005), and are more heterogeneous than the Japanese calligraphies used by Gershoni and Hochstein (2011). Because stimulus complexity is an important factor for aesthetic appreciation (Leder, Belke, Oeberst, & Augustin, 2004; Palmer, Schloss, & Sammartino, 2013; Tinio & Leder, 2009), we also applied a corresponding objective measure.

In two experiments, we collected three different ratings for each of the pictures: perceptual balance, liking, and stability. In our first experiment, we could not find a significant correlation between balance and liking ratings. However, liking was strongly related to complexity. After discounting this effect, a reliable relationship between balance and liking ratings emerged.

However, our objective measures of perceptual balance did still not show a significant correlation with the ratings. Given these results, we reasoned that for VAST images there might be a different concept of perceptual balance that is not related to our objective measures of balance. Therefore, in our second experiment, we considered an alternative concept of balance.

## Experiment 1

In this experiment, we wanted to further investigate the relationship between perceptual balance, aesthetic appreciation, and balance-related measures. As stimulus set we used pictures from the VAST (Götz, 1981). This test consists of 42 pairs of nonrepresentational gray-level pictures. The pictures in each pair are the same, except that one is better configured than the other. Usually, the pictures are used for measuring aesthetic sensitivity. The person under examination has to decide which picture of each pair is the “correct” one. For the present objective, though, we applied only the 42 correct pictures as stimuli, and our participants had to rate each picture with respect to liking and perceptual balance.

The VAST (Götz, 1981) pictures are more complex and more diverse than those applied in several former studies on perceptual balance (e.g., Fillinger & Hübner, 2018; Gershoni & Hochstein, 2011; Hübner & Fillinger, 2016; Wilson & Chatterjee, 2005), but are still less complex than most of real artworks. To take the variation in complexity into account, we also wanted to have objective scores for this property. An often used measure in this respect is the Kolmogorov complexity, which is deﬁned as the length of the shortest program that can describe an item (Donderi, 2006), and can be approximated by the jpeg compression algorithm (Faloutsos & Megalooikonomou, 2007). Therefore, we used the corresponding ﬁle sizes of the images to construct complexity scores.

We expected that the balance scores reliably predict perceptual balance ratings as well as liking ratings. Furthermore, in addition to balance, complexity should also affect liking (e.g., Tinio & Leder, 2009).

### Method

#### Participants

Fifty-two persons (16 male, mean age 24.6 years, *SD* = 7.95) participated in the rating task and received a 3-€ voucher as incentive. They were recruited via a local online system (ORSEE; Greiner, 2015). This study was carried out in accordance with the ethical guidelines of the Universität Konstanz, which is based on the Declaration of Helsinki. Participants were informed of their right to abstain from participation in the study or to withdraw consent to participate at any time without reprisal.

#### Stimuli

As stimuli, we used the 42 correct pictures from the VAST (Götz, 1981). Corresponding images were created by digitizing (264 × 330 pixels) a paper version of the VAST, which was ordered from our library. The copyright for the works of Karl Otto Götz are held by VG Bild-Kunst, Germany. Thumbnails of the images can be seen in Table 1. The images were positioned at the center of the screen on a gray background. Stimulus presentation and response registration, which occurred exclusively online, were controlled by the SoSci Survey System (Leiner, 2016).

**Table 1.** Results of Experiment 1.

	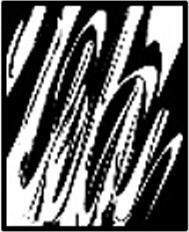	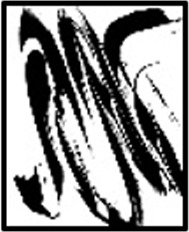	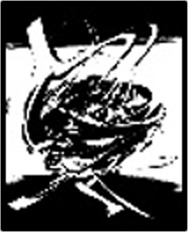	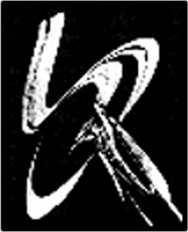	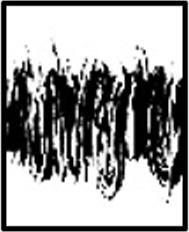	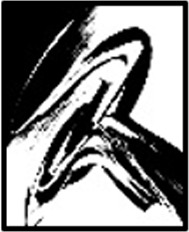	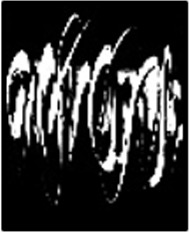	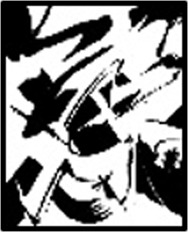	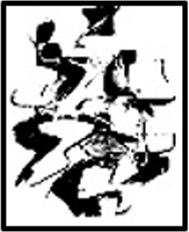	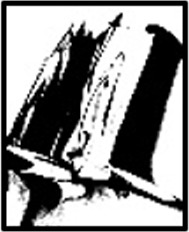	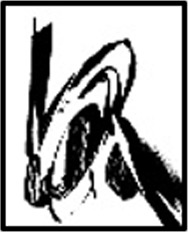	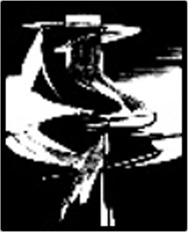	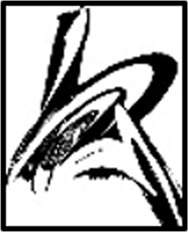	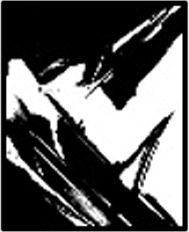
**#**	**33**	**24**	**16**	**4**	**39**	**26**	**11**	**30**	**42**	**5**	**20**	**36**	**41**	**12**
C	*0.54*	*0.47*	*0.46*	*0.46*	*0.46*	*0.42*	*0.41*	*0.40*	*0.40*	*0.37*	*0.35*	*0.35*	*0.35*	*0.33*
L	63	57	58	63	61	60	62	54	59	55	54	60	54	54
B	58	52	56	46	73	46	68	44	55	37	37	48	43	34
	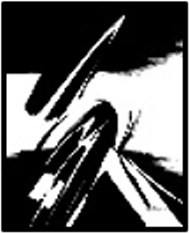	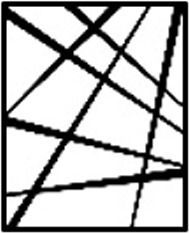	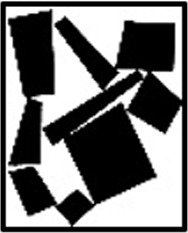	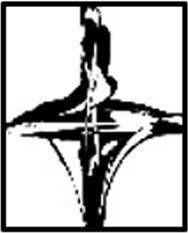	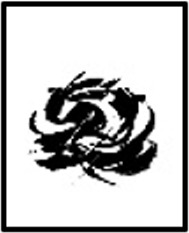	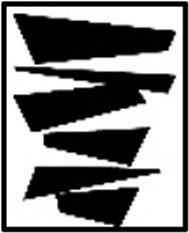	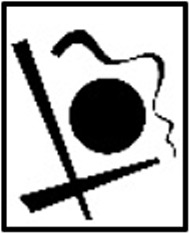	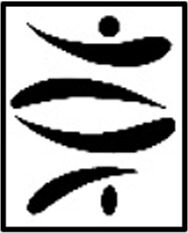	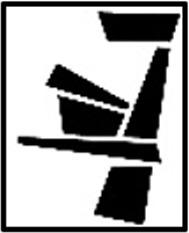	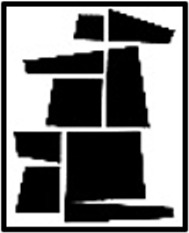	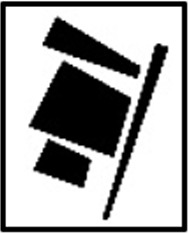	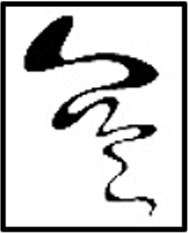	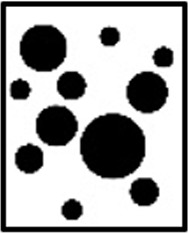	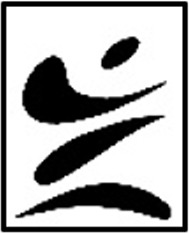
**#**	**10**	**19**	**7**	**22**	**8**	**37**	**15**	**3**	**23**	**29**	**18**	**9**	**25**	**34**
C	*0.31*	*0.30*	*0.25*	*0.24*	*0.22*	*0.22*	*0.20*	*0.19*	*0.19*	*0.19*	*0.18*	*0.17*	*0.17*	*0.17*
L	59	57	31	59	62	38	40	57	32	40	32	50	42	46
B	38	57	39	75	68	60	40	72	37	56	33	48	56	58
	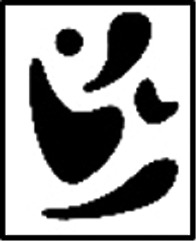	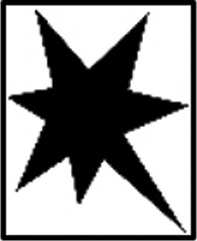	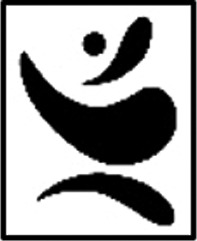	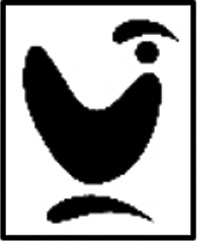	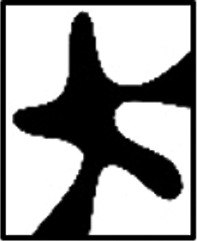	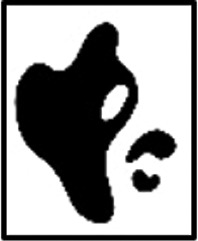	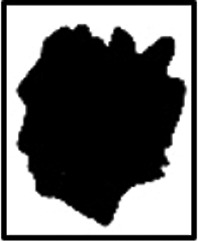	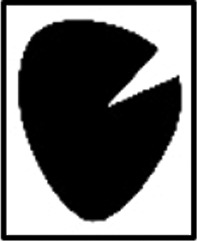	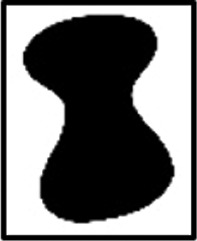	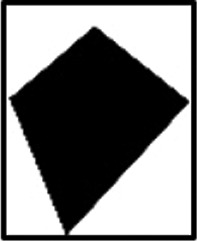	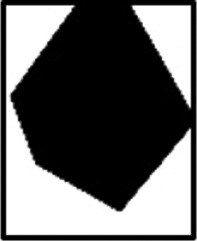	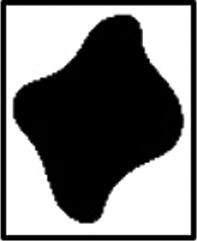	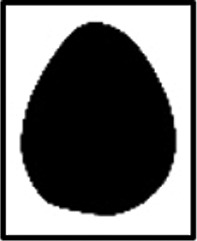	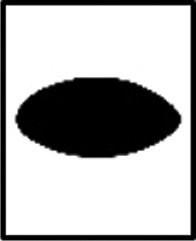
**#**	**38**	**14**	**2**	**1**	**35**	**40**	**17**	**28**	**27**	**31**	**6**	**21**	**32**	**13**
C	*0.16*	*0.16*	*0.16*	*0.15*	*0.14*	*0.13*	*0.12*	*0.11*	*0.11*	*0.11*	*0.10*	*0.10*	*0.10*	*0.08*
L	45	36	52	48	38	37	29	27	30	27	22	27	25	27
B	53	45	61	54	46	36	53	33	64	45	45	59	71	76

The thumbnails of the VAST pictures, shown in the continuing first row, are ordered by complexity. The corresponding scores are given in row 3(C). The bold numbers in row 2(#) represent the corresponding picture numbers. Mean ratings for liking (underlined) and balance are shown in the third (L) and fourth (B) row, respectively. (For the works of Karl Otto Götz: © VG Bild-Kunst, Bonn 2017.)

#### Objective Measures

We computed the APB and DCM scores for each picture as objective measures of balance; see Hübner and Fillinger (2016) for details of calculation. The DCM scores ranged from 1.82 to 26.9 (*M* = 9.85, *SD* = 6.56), and the APB scores from 5.53 to 47.0 (*M* = 21.8, *SD* = 8.62). As measure of complexity, we used the ratio between the file size of the jpeg-compressed image and that of the uncompressed image (Palumbo, Ogden, Makin, & Bertamini, 2014), where a value close to 1 indicates a very complex image and a value close to 0 a very simple one. In our stimulus set, complexity varied between 0.08 and 0.54 (*M* = 0.25, *SD* = 0.13).

#### Procedure

The experiment, which was carried out exclusively online, started with an instruction that informed the participants about the task (the instruction in German and its English translation are provided in Appendix B). In addition, we used a seriousness check (Reips, 2009) to control for participants’ involvement in the task. Next, there were two blocks in each of which the 42 stimuli were presented in random order. In the first block, the participants had to rate how much they liked the stimuli (from *I do not like it* to *I like it*), and in the second block how well the stimuli were balanced (from *not balanced* to *balanced*). Internally, the scale ranged from 0 to 100 (values not visible for the participants). The ratings were entered by clicking with the mouse on a continuous slider. Immediately after each response, the next stimulus was displayed. Altogether, the experiment comprised 84 trials (2 × 42 trials) and lasted about 10 minutes.

### Results

All 52 participants passed the seriousness check. Mean balance and liking ratings were 51.8 (*SD* = 27.4) and 45.9 (*SD* = 28.2), respectively. The means across participants for each picture are shown in Table 1. Correlational analyses revealed that overall there was no significant correlation between the two ratings (see Table 2). Concerning the relations with the objective measures, there was merely a significant and strong correlation between liking and complexity, indicating that the more complex a picture was, the more it was liked.

**Table 2.** Correlations^a^ Between Stability, Balance, and Liking Ratings (Means Across Stimuli) and Objective Measures Separated for Every Visual Category.

		Balance_2_	Liking	Stability	Complexity	APB	DCM
Balance_1_	Overall (*df* = 40)	0.928***	0.154	0.524***	–0.048	0.108	–0.265
Balance_2_		–	0.086	0.601***	–0.136	0.059	–0.305
Liking		0.086	–	–0.535***	0.809***	–0.261	0.274
Stability		0.601***	–0.535***	–	–0.639***	0.262	–0.236
Balance_1_	Dynamic pattern (*df* = 15)	0.921***	0.592*	0.582*	0.223	0.232	–0.200
Balance_2_		–	0.597*	0.489*	0.172	0.124	–0.214
Liking		0.597*	–	0.406	0.486*	0.084	0.148
Stability		0.489*	0.406	–	–0.055	0.325	0.152
Balance_1_	Multiple elements (*df* = 13)	0.951***	0.844***	0.669**	0.186	–0.258	–0.328
Balance_2_		–	0.805***	0.799***	0.268	–0.311	–0.319
Liking		0.805***	–	0.664**	0.236	–0.179	–0.267
Stability		0.799***	0.664**	–	0.298	–0.248	–0.033
Balance_1_	Single element (*df* = 8)	0.922***	–0.228	0.802**	–0.541	0.384	–0.625
Balance_2_		–	–0.066	0.731*	–0.411	0.377	–0.754*
Liking		–0.066	–	–0.464	0.830**	0.151	–0.044
Stability		0.731*	–0.464	–	–0.593	0.466	–0.313

*Note*. *df =* degrees of freedom; APB = Assessment of Preference for Balance; DCM = Deviation of the Center of “Mass.”

^a^For many of the correlations in this table we had a specific hypothesis. Therefore, we did not correct for multiple testing. When interpreting other significant correlations, however, one should be aware of the problem of inflating false-positive rates with multiple testing.

*p < 0.05. **p < 0.01. ***p < 0.001.

To get an idea in what sense complexity varies across the VAST pictures, we ordered the pictures accordingly. As can be seen in Table 1, complexity seems to be largely determined by the number of elements in a picture. Moreover, the order suggests that there are three categories of pictures. The 10 pictures with the least complexity contain only one element, whereas the following 17 pictures of medium complexity are composed of multiple elements. The remaining 15 pictures of highest complexity consist of countless elements, and also represent some dynamics or implied motion.

**Figure 1.**
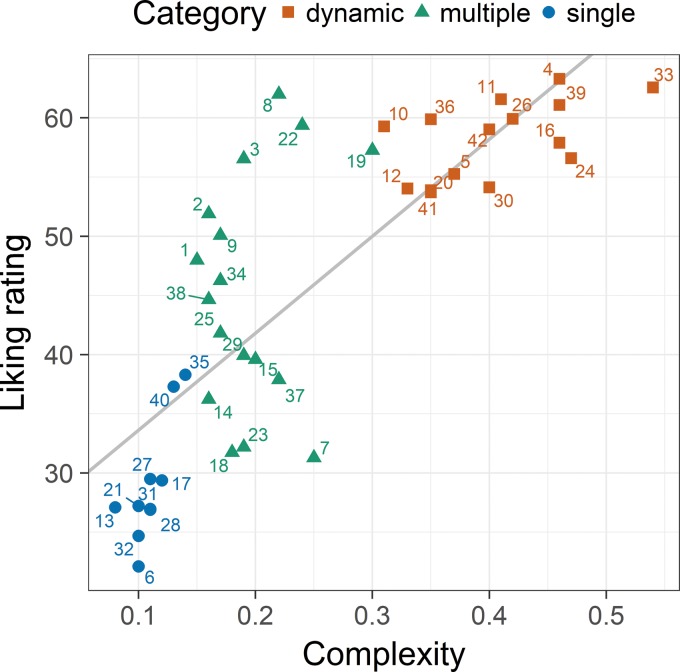
Relation between complexity (ratio between the file size of the jpeg-compressed image and that of the uncompressed image) and liking ratings in Experiment 1 and the corresponding linear regression line. Each data point represents the picture according to its number (see Table 1). The colors and shapes of the symbols indicate the corresponding preliminary category.

The relation between complexity and liking is also shown in Figure 1, where the three suggested picture categories are indicated by specific shapes and colors of the corresponding data points. In view of this result, an interesting further question was to what extent the relations between the ratings and measures differ between the categories. However, before we tried to answer this question, we first wanted to test whether the assumed categories are common sense, and if so, exactly which pictures are assigned to each category.

#### Categorization Check

**Figure 2.**
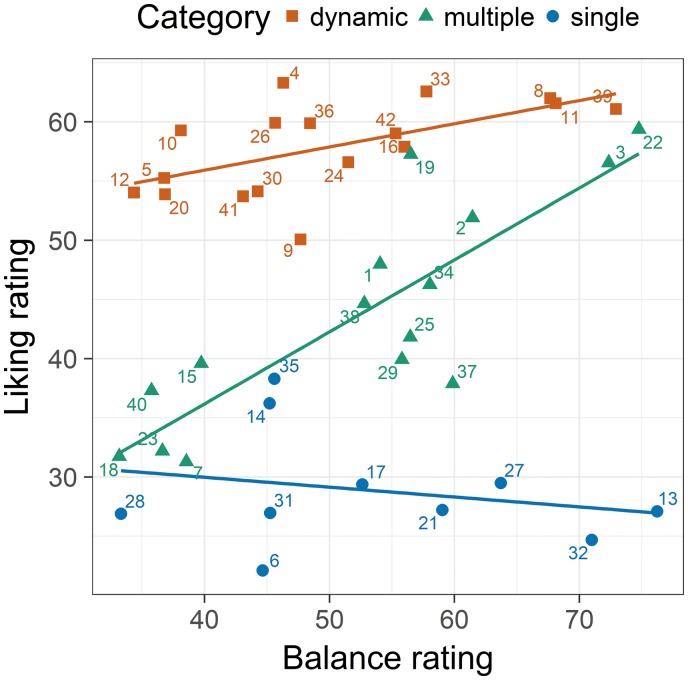
The relation between balance ratings and liking ratings in Experiment 1 for the different picture categories obtained in the supplementary categorization study. Each data point represents the picture according to its number (see Table 1). The colors and shapes of the symbols indicate the corresponding category. The lines are the corresponding regression lines

In order to examine how people spontaneously categorize the VAST pictures, we conducted a supplementary visual categorization study. The task of the participants was to sort each picture into one of three nonlabeled categories. The detailed method and results are provided in Appendix A. Here, it is sufficient to know that the majority of participants confirmed our overall categorization. Merely four of the pictures (nos. 40, 14, 8, and 9) were categorized differently relative to the categorization shown in Figure 1 (see Figure 2). In view of this result, we decided to name the three empirically validated categories “single element,” “multiple elements,” and “dynamic pattern,” respectively.

The mean ratings for pictures in these categories are: single element (balance 53.7, *SD* = 13.5; liking 28.8, *SD* = 4.95), multiple elements (balance 52.4, *SD* = 13.0, liking 43.7, *SD* = 9.36), and dynamic pattern (balance 50.0, *SD* = 11.6, liking 57.9, *SD* = 3.84). One-way ANOVAs revealed that the balance ratings did not differ between the categories, *F*(2, 39) = 0.291; *p* = 0.749; η^2^ = 0.015. However, liking differed significantly, *F*(2, 39) = 62.9; *p* < 0.001; η^2^ = 0.763.

The correlations between the ratings and measures for each individual category are shown in Table 2. As can be seen, single-element pictures were not only liked least, but their aesthetic appreciation was also independent of perceptual balance. In contrast, for pictures with multiple elements and for those with dynamic patterns, there was a significant correlation between liking and perceptual balance. The more the pictures were perceived as balanced, the more they were liked, where the relation was more pronounced for the multiple-element pictures.

Finally, it should be noted that, although there was a strong correlation between liking and complexity for the whole stimulus set, within categories such a correlation was reliable only for the dynamic-pattern and single-element pictures (see Table 2).

### Discussion

In this experiment, we investigated the relationship between perceptual balance, aesthetic appreciation, and related objective measures. As stimuli, we presented pictures from the VAST (Götz, 1981; Götz et al., 1979). A first data analysis revealed that, overall, balance ratings did not correlate with liking ratings. However, liking strongly depended on the complexity of the pictures. Overall, the more complex a picture was, the more it was liked, which is in line with former results (e.g., Jacobsen, 2004; Tinio & Leder, 2009). Moreover, when we ordered the pictures by complexity, it became obvious that complexity increased with the number of elements in a picture. Already Berlyne (1971) observed such a relation (see also Nadal, Munar, Marty, & Cela-Conde, 2010). Interestingly, visual inspection suggested that the VAST pictures can easily be divided into three categories: single-element, multiple-element, and dynamic-pattern pictures. In a supplementary categorization study (Appendix A), we confirmed and refined our preliminary categorization.

The categorization of the pictures offered the possibility to analyze the relations between the variables separately for each category. As a result, we found that perceptual balance and liking correlated significantly for multiple-element and dynamic-pattern pictures, but not for single-element ones. This indicates that the effect of perceptual balance on liking depends on the picture type. More specifically, balance seems to be an important factor for liking only for pictures with a sufficient number of elements or complexity.

However, despite these positive relations, the formal measures of balance (APB and DCM) were unrelated to perceptual balance and liking. This held across all pictures as well as within each category and indicates that the concept of balance reflected by these measures is different from that applied by our participants.

Concerning complexity, despite the strong overall correlation with liking, within categories, it was reliable only for dynamic-pattern and single-object pictures. If we consider the order of these pictures with respect to complexity (Table 1), then it seems that complexity increases with the number of edges, which is in line with Berlyne (1971), who also observed that irregularity of shape affects complexity. Recently, Friedenberg and Bertamini (2015) showed that the preference increases with contour length and the number of concavities.

Taken together, the results of this experiment demonstrate that the relationship between balance and liking depends on the picture type. Furthermore, the fact that the balance ratings were unrelated to the formal measures of balance, even for those picture categories where balance and liking were correlated, shows that a different concept of perceptual balance was applied. But which property was used by our participants for assessing the balance of a picture? If one inspects the multiple-element pictures (Table 1), then it seems that at least in some pictures rated as well balanced, the elements are piled up in a more stable way than in those rated as less balanced. Even for the dynamic-pattern pictures, it appears that the most balanced ones look more stable than the less balanced ones.

Therefore, we hypothesized that stability could have been applied by our participants as concept of balance. To test whether this was the case, we conducted a further experiment, in which we collected stability ratings for the VAST pictures.

## Experiment 2

In the previous experiment, we observed that perceptual balance correlated for some picture types with liking, but was generally unrelated to the formal measures of balance. This indicates that the participants applied some concept of balance that was different from that reflected by the measures. A possible alternative in this respect might be stability. As mentioned in the Introduction, already Pierce (1894) observed that perceptual stability is a variant of balance, especially for vertically arranged elements in a picture. More recently, Friedenberg (2012) even proposed a *perceptual instability* hypothesis, stating that objects perceived as more fragile are less attractive.

Perhaps our participants rated the balance of the VAST pictures, at least the more complex ones, by assessing the compositions’ stability. In order to test whether balance was indeed interpreted as stability in Experiment 1, we asked the participants in this experiment to rate stability directly. If our supposition is correct, then stability ratings should highly correlate with balance as well as with liking ratings, at least for multiple-element and dynamic-pattern pictures.

Because the participants in Experiment 1 rated balance after they had assessed the aesthetic appreciation of the pictures, there might have been some carryover effects from liking to balance ratings. Therefore, in the present experiment, we again collected balance ratings for the VAST pictures, but this time from an independent sample of participants.

### Method

Altogether, 104 (20 male, mean age 24.0 years, *SD* = 5.10) persons participated in the online rating tasks. As incentive, each participant had the chance to win one of fifty 3-€ vouchers. The experiment was similar to the first one, except that half of the participants (9 male, mean age 24.2 years, *SD* = 5.72) rated the picture with respect to balance (from *not balanced* to *balanced*), whereas the other half (11 male, mean age 23.8 years, *SD* = 4.45) rated how stable the composition of the pictures was (from *unstable* to *stable*). The instruction is provided in Appendix B. Each task lasted about 5 minutes.

### Results

All 104 participants passed the seriousness check. The mean balance rating was 53.0 (*SD* = 11.1), and the mean stability rating 50.8 (*SD* = 13.2). A one-way ANOVA revealed that there was again no significant difference between the balance ratings for the different picture categories (single element: *M* = 55.5, *SD* = 11.4; multiple elements: *M* = 54.8, *SD* = 12.1; dynamic pattern: *M* = 50.0, *SD* = 9.78), *F*(2, 39) = 1.08; *p* = 0.351; η^2^ = 0.052. Moreover, the balance ratings were rather similar to those in Experiment 1 (*r*(40) = 0.928, *p* < 0.001). Accordingly, the present balance ratings correlated similarly strong as the previous balance ratings with the liking ratings from Experiment 1 (see Table 2).

This time, the DCM scores correlated significantly with the balance ratings, but only for single-element pictures. The corresponding correlation in Experiment 1 was already relatively high, but shortly failed to reach significance. Because the correlation between liking and the present balance ratings is reduced, compared to Experiment 1, the increased correlation with the DCM scores could indicate that the former balance ratings were indeed biased to some extent.

**Figure 3.**
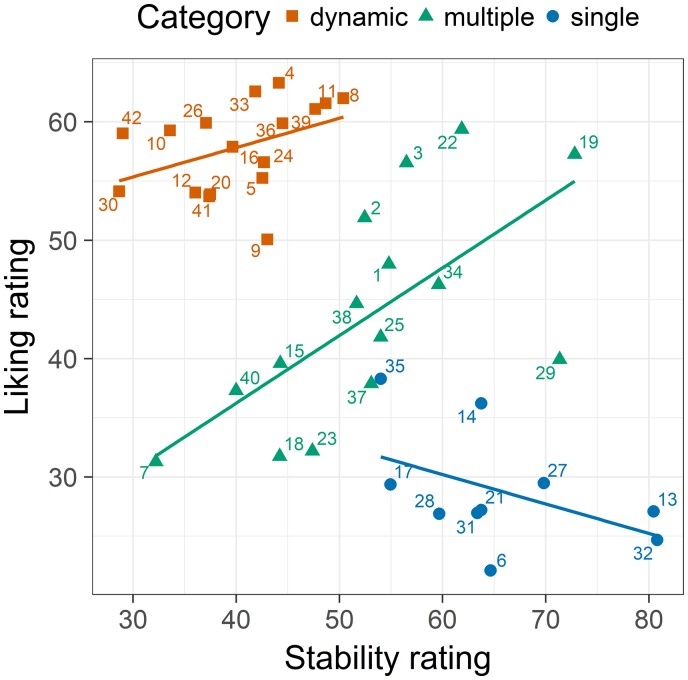
The relations between stability ratings and liking ratings from Experiment 1 for the categories obtained in the supplementary categorization study. Each number represents 1 of the 42 stimuli. The colors indicate the stimulus categories. The lines represent the regression lines.

Other than the balance ratings, stability ratings differed significantly between the categories (single element: *M* = 65.5, *SD* = 9.22; multiple elements: *M* = 53.1, *SD* = 10.9; dynamic pattern: *M* = 40.3, *SD* = 6.29), *F*(2, 39) = 26.4; *p* < 0.001; η^2^ = 0.575. Accordingly, the range of stability between the categories differed (Figure 3). Dynamic-pattern pictures were rated low and single-element stimuli high in stability, whereas the multiple-element pictures covered almost the entire range of stability. Together, the different ranges produced a negative overall correlation between stability and liking (Table 2). Given this result, it is not surprising that stability also correlated negatively with complexity. Thus, complex stimuli were rated low in stability. Nevertheless, they were much liked.

Analyses of the relations within the different stimulus categories show that for multiple-element pictures there was a significant positive relation between stability and liking (Table 2). The more stable the pictorial arrangement was rated, the more it was liked. However, multiple regression revealed that the stability ratings do not explain variance that is not already explained by balance, β = .056, *t*(12) = 0.196, *p* = 0.848. This indicates that at least for multiple-element pictures, balance and stability ratings were based on a rather similar concept.

### Discussion

In this experiment, we collected stability and balance ratings for the VAST pictures. Our aim was to test whether the balance ratings in Experiment 1 were accomplished by assessing the pictures’ stability. If that were the case, then stability ratings should not only be highly correlated with the balance ratings but also with the liking ratings for multiple-element and dynamic-pattern pictures. Such a result would explain why the formal balance measures (DCM and APB) did not correlate with the balance ratings in the previous experiment. They simply reflect a different concept of balance.

If we consider the results, then it is obvious that our hypothesis was only partially confirmed. Although balance and stability ratings correlate significantly, their relation was far from perfect. Both variables share only about 36% of their variance, which indicates that the two concepts are related but not identical. This conclusion is also confirmed by the negative overall correlation between stability and liking. The dynamic patterns, which were liked most, were rated as relatively unstable. In contrast, single-element pictures, which were liked least, were rated as most stable. Only the multiple-element pictures cover the whole range of stability. Moreover, for these pictures, stability was positively related to liking, as was the case for balance. For single-element pictures as well as for the dynamic-pattern pictures liking was independent of stability.

Because of the within-participants design in Experiment 1, we speculated that there could have been carryover effects from liking to balance ratings. Therefore, we again collected balance ratings, but this time with a between-participants design. The high correlation between the two balance ratings indicates that carryover effects, if present at all, were rather small. Nevertheless, the independent balance ratings for the single-element pictures now correlated negatively with the DCM scores, which means that the pictures were perceived as more balanced the less their center of “mass” deviated from their geometrical center.

Taken together, the results of this experiment demonstrate that balance and stability ratings for the VAST pictures are related, but not identical. Across all pictures, there was a negative correlation between stability and liking, whereas balance was unrelated to liking. If one considers the relations within each picture type, then it seems that the participants performed the rating tasks differently, depending on the stimulus type. If pictures contained multiple elements, then balance was interpreted in the same way as stability. Accordingly, both variables were positively correlated with liking. However, for the other stimulus types, balance ratings and stability ratings were conceptualized and performed differently.

## General Discussion

The objective of this study was to further investigate the relation between perceptual balance and aesthetic appreciation. It has widely been assumed that the aesthetic appreciation of a picture depends on how well it is balanced, where balance has often been defined analogously to mechanical balance (e.g., Arnheim, 1954; Pierce, 1894; Puffer, 1903). Recently, even objective balance measures have been proposed, like the DCM (Hübner & Fillinger, 2016; McManus et al., 2011), or the APB (Wilson & Chatterjee, 2005), which more or less also rely on the mechanical-balance metaphor. Although these measures have successfully been applied to predict balance and liking ratings, the considered pictures were mostly relatively simple. Therefore, an important question is to what extent these concepts and measures are also valid for more complex pictures. Up to now, the provided evidence in this area is equivocal.

Whereas in some studies the measures could successfully predict the aesthetic appreciation of various photographs (McManus et al., 2011; Thömmes & Hübner, 2018), there are also negative results. Gershoni and Hochstein (2011), for instance, examined Japanese calligraphies and found no correlation between APB scores and balance ratings. In a recent study with the same stimuli, we could show that if prototypicality is taken into account, then at least the DCM is related to liking, but only for less prototypical calligraphies (Fillinger & Hübner, 2018).

These results demonstrate that the relationships between aesthetic appreciation, perceptual balance, and liking are still largely unknown, especially for more complex pictures. Therefore, for the present experiments, we used pictures from the VAST (Götz, 1981; Götz et al., 1979). These stimuli are more complex than those applied in the studies that introduced the formal balance measures, but are still less complex than photographs or most of real artworks.

In Experiment 1, the participants had first to rate how much they liked the VAST pictures and then to assess how well these pictures are balanced. As a result, across all pictures, there was no correlation between these two variables. This is similar to our result with Japanese calligraphies (Fillinger & Hübner, 2018). However, we found that the liking of the VAST pictures depended strongly on complexity, which is in line with other results (e.g., Berlyne, 1971; Tinio & Leder, 2009). When we inspected the pictures more closely, then it became clear that complexity was largely determined by the number of elements in the pictures (see also Jacobsen, 2004; Nadal et al., 2010). Even more, the VAST pictures could be divided into single-element, multiple-element, and dynamic-pattern pictures, the latter with countless elements. This categorization was confirmed and refined in a supplementary categorization study (Appendix A).

Therefore, in a next step we analyzed the relations between ratings and scores separately for each category and found that for single-element pictures, which were liked least, perceptual balance had no effect on liking. However, for dynamic-pattern pictures, which were liked most, liking increased significantly with perceptual balance. The numerically largest correlation, though, occurred for the multiple-element pictures, which makes sense, because balancing should play some role if a medium number of elements are present in a picture. These findings are in line with the principle of *unity-in-variety* (Fechner, 1876) and the related idea that complexity must go along with order for aesthetic appreciation (Van Geert & Wagemans, 2019). Accordingly, balance affects liking only when an image is sufficiently complex, which, in our case, is the case for dynamic-pattern and multiple-element pictures, but not for single-element pictures. Thus, the results from our first experiment demonstrate that perceptual balance has a positive effect on aesthetic appreciation, but only for certain types of pictures.

Despite these positive relations, there were no reliable correlations between the ratings and the applied formal measures of balance, which indicates that the participants used a concept of balance that was different from that reflected by the APB and DCM scores. A closer look at the multiple-element pictures suggested that visual (gravitational) stability could be a promising candidate as an alternative concept of balance. That stability affects liking, especially for vertically arranged picture elements, has already been proposed by Pierce (1894). Stability could even have been relevant for the dynamic-pattern and single-element pictures.

To test whether our participants indeed rated the balance of the pictures by assessing their stability, we conducted a second experiment where we asked our participants directly to rate the stability. As a result, stability correlated significantly with balance, but not very high. Moreover, across all stimuli, stability was negatively correlated with liking. This was due to the fact that, different from balance, the range of stability differed systematically between the different stimulus categories. The most complex pictures, that is, the dynamic patterns, which were liked most, were rated as least stable, whereas the least complex (single-element) pictures, which were liked least, were judged as the most stable. Within categories, stability was related to liking only for the multiple-element pictures. For these stimuli, the correlation was positive, as expected, that is, the more stable a configuration, the more it was liked. Because a similar relation held for the balance ratings, it can be assumed that for this picture type balance was interpreted in the sense of stability and vice versa. In any case, balance was not interpreted in the sense reflected by the formal balance measures.

In Experiment 2, we again collected balance ratings, but this time with a between-participants design. As a result, for the single-element pictures, there was a reliable correlation between the balance ratings and the DCM scores, which indicates that pictures were rated as more balanced if the center of mass was closer to the geometric center of the picture.

Thus, it seems that the concept of stability was not used consistently. Across all stimuli, the corresponding ratings correlated negatively with liking, whereas they correlated positively within the multiple-element pictures. How can this discrepancy be explained? Because stability should actually have a positive effect on aesthetic appreciation (Friedenberg, 2012; Pierce, 1894), we think that the negative overall correlation is due to a confound with other variables. The result that the dynamic patterns were liked most is probably not due to their low stability, but to the fact that in this specific case instability goes along with implied motion, which is usually liked. Interestingly, for Japanese calligraphies, we observed a similar result (Fillinger & Hübner, 2018). At least atypical calligraphies were liked the more, the less stable they were. As shown by Dubal et al. (2014), dynamic brushstrokes in calligraphies convey emotions (Dubal et al., 2014). Thus, it seems that instability has no negative effects on liking, if it results from a dynamic that implies motion associated with positive emotion.

Moreover, the result that single-element pictures were liked least is presumably not due to their great stability, but rather to their restricted variety.

Thus, it seems that three types of perceptual balance/stability occurred in our study. For assessing the balance of the single-element pictures, the participants applied mechanical balance, that is, the deviation of the center of “mass” from the geometrical center, as reflected by the DCM. However, this balance was unrelated to liking. For rating the balance of the multiple-element pictures, the gravitational stability of the configuration was assessed, which was positively associated with liking. Finally, the low stability ratings of the dynamic patterns were presumably not due to their perceived low gravitational stability, but to their dynamics and implied motion. The corresponding high emotional expressivity led these pictures to be liked most.

Taken together, our study shows that perceptual balance and aesthetic appreciation are related in a complex way. How balance is interpreted and assessed depends largely on the content of the picture. Moreover, other factors such as complexity can be dominant or at least modulate the relation between balance/stability and liking. The more factors are known and, therefore, can be discounted, the better the pure effect of balance can be isolated.

## Authors’ Note

The data of both experiments are available at DOI OSF.IO/4BUQW.

## Appendix A

The supplementary study reported here served to confirm and refine our preliminary categorization of the VAST pictures based on complexity and the number of elements.

### Method

Twenty-one persons (12 male, mean age 25.7 years, *SD* = 7.80) from the Universität Konstanz participated and received goodies for their contribution. None of the persons had participated in Experiment 1. The task was performed under the same ethical standards as the main experiment.

### Stimuli and Procedure

The digitized 42 pictures from the VAST (Götz, 1981) were printed on white cardboards (12 × 15 cm). The participants were first asked to spread all cards in front of them and then to sort the 42 cards into three categories by the pictures’ features within approximately 1 minute. Subsequently, they were interviewed about the features they had used for categorization. In total, the session lasted about 5 minutes.

### Results and Discussion

Fifteen of the 21 participants used the same stimulus features. Given the resulting categories, we assigned each picture to the category chosen most. A multipattern *χ^2^*-Test (3 categories × 42 stimuli) revealed that the distribution of frequencies across categories was not random, *χ^2^*(82) = 906.90, *p* < 0.001, *V* = 0.188. The assignments were slightly different from our preliminary ones. Picture #40 was sorted into the single-element category instead of the multiple-element category. For Picture #14 it was the other way round. Furthermore, Picture #8 and Picture #9 were assigned to the dynamic-pattern category instead of the multiple-element category.

In the single-element category, the APB score ranged from 18.7 to 38.8 (*M* = 25.2, *SD* = 6.22), the DCM score from 1.52 to 13.8 (*M* = 5.65, *SD* = 3.98), and complexity from 18 to 38 (*M* = 26.5, *SD* = 5.56). Moreover, the APB score ranged from 5.64 to 34.5 (*M* = 22.6, *SD* = 8.21), the DCM score from 3.20 to 26.4 (*M* = 10.0, *SD* = 6.50), and complexity from 32 to 71 (*M* = 45.8, *SD* = 10.4) for multiple-element pictures. Finally, in the dynamic-pattern category, the APB score ranged from 5.53 to 47.0 (*M* = 19.3, *SD* = 9.76), the DCM score from 5.47 to 68.3 (*M* = 14.9, *SD* = 15.2), and complexity from 41 to 127 (*M* = 90.5, *SD* = 21.7).

The pictures in the resulting single-element category were described by the participants as, for example, “clear shapes consisting of a big black area,” “single coherent elements,” or “chunky elements.” The multiple-element pictures were described as “several black elements,” “single shapes scattered all over,” or “multiple elements.” The dynamic-pattern category was described, for instance, as “abstract art,” “complex patterns made by brushstrokes,” or “movements.” The other six participants also used the dynamic-pattern category, but divided the other pictures into two categories that can be labeled as “curved elements” and “angular elements.”

Thus, by and large, this study confirmed our preliminary categorization. The majority of the participants used the number of elements and the picture’s dynamics for their categorization.

### Appendix B

In this part, we provide the instructions (originally in German) and their English translations.

#### Experiment 1

##### Liking ratings

On the following pages, you will see simple pictures. Your task is to rate these images according to certain criteria. The survey consists of two parts. In the first part, you should rate how much you like the pictures. Make your decisions spontaneously! After you have decided, set the slider between “I do not like it” and “I like it” and click on “Next” to go to the next page. Since these are subjective assessments, there are no right or wrong answers.

[In German (original): Auf den folgenden Seiten werden Sie einfache Bilder sehen. Ihre Aufgabe besteht darin, diese Bilder nach gewissen Kriterien zu beurteilen. Die Befragung besteht aus zwei Teilen. Im ersten Teil sollen Sie beurteilen, wie sehr Ihnen die Bilder gefallen. Treffen Sie Ihre Entscheidungen ganz spontan! Nachdem Sie sich entschieden haben, stellen Sie den Regler zwischen “gefällt mir gar nicht” und “gefällt mir sehr gut” ein und klicken auf “Weiter” um zur nächsten Seite zu gelangen. Da es sich um subjektive Einschätzungen handelt, gibt es keine richtigen oder falschen Antworten.]

##### Balance ratings

In the second part of the survey, you will rate the images with regard to balance. Set the slider between “not balanced” and “balanced.”

[In German (original): Im zweiten Teil der Befragung sollen Sie die Bilder nun in Hinsicht auf Balance beurteilen. Stellen Sie hierzu den Regler zwischen “nicht balanciert” und “balanciert” ein.]

#### Experiment 2

##### Stability ratings

On the following pages, you will see simple pictures. Your task is to rate how stable the arrangement of the picture elements appears to you. Make your decisions spontaneously! After you have decided, set the slider between “unstable” and “stable” and click on “Next” to go to the next page. Since these are subjective assessments, there are no right or wrong answers.

[In German (original): Auf den folgenden Seiten werden Sie einfache Bilder sehen. Ihre Aufgabe besteht darin, zu beurteilen, wie stabil Ihnen die Anordnung der Bildelemente erscheint. Treffen Sie Ihre Entscheidungen ganz spontan! Nachdem Sie sich entschieden haben, stellen Sie den Regler zwischen “instabil” und “stabil” ein und klicken auf “Weiter” um zur nächsten Seite zu gelangen. Da es sich um subjektive Einschätzungen handelt, gibt es keine richtigen oder falschen Antworten.]

##### Balance ratings

On the following pages, you will see simple pictures. Your task is to rate them in terms of balance. Make your decisions spontaneously! After you have decided, set the slider between “not balanced” and “balanced” and click on “Next” to go to the next page. Since these are subjective assessments, there are no right or wrong answers.

[In German (original): Auf den folgenden Seiten werden Sie einfache Bilder sehen. Ihre Aufgabe besteht darin, diese in Hinsicht auf Balance zu beurteilen. Treffen Sie Ihre Entscheidungen ganz spontan! Nachdem Sie sich entschieden haben, stellen Sie den Regler zwischen “nicht balanciert” und “balanciert” ein und klicken auf “Weiter” um zur nächsten Seite zu gelangen. Da es sich um subjektive Einschätzungen handelt, gibt es keine richtigen oder falschen Antworten.]
